# Editorial Expression of Concern: mtDNA structure: the women who formed the Brazilian northeast

**DOI:** 10.1186/s12862-025-02366-y

**Published:** 2025-03-21

**Authors:** Ana Paula Schaan, Lorenna Costa, Diego Santos, Antonio Modesto, Marcos Amador, Camile Lopes, Sílvia Helena Rabenhorst, Raquel Montenegro, Bruno D. A. Souza, Thayson Lopes, France Keiko Yoshioka, Giovanny Pinto, Vivian Silbiger, Ândrea Ribeiro-dos-Santos

**Affiliations:** 1 Human and Medical Genetics Laboratory, Federal University of Pará, Av. Augusto Corrêa, 01– Cidade Universitária Prof. José Silveira Netto– Guamá, Belém, 66075-110 PA Brazil; 2 Clinical and Toxicological Analyses Department, Federal University of Rio Grande do Norte, Natal, 59300-000 RN Brazil; 3 Pathology and Legal Medicine Department, Federal University of Ceará, Fortaleza, 60020-181 CE Brazil; 4 Genetics and Molecular Biology Laboratory, Federal University of Piauí, Parnaíba, 64202-020 PI Brazil; 5 Center of Oncological Research, Federal University of Pará, Belém, 66073-005 PA Brazil; 6 Center of Research and Drug Development, Federal University of Ceará, Fortaleza, 60430-270 CE Brazil

**Editorial Expression of Concern**: *BMC Evol Biol***17**, 185 (2017)

10.1186/s12862-017-1027-7

The Editor is issuing an editorial Expression of Concern for this article because the methodology exhibits an absence of documented quality control (QC) measures, the use of unpublished software (AMBASE) and a lack of transparency regarding manual haplogroup curation. Given these limitations readers are therefore recommended to interpret the results and conclusions with caution. The authors are reanalysing their data and appropriate editorial action will be taken when this is complete. Ana Paula Schaan, Giovanny R Pinto, Ândrea Ribeiro-dos-Santos and France Keiko Yoshioka have agreed to this statement. Lorenna Costa, Diego Santos, Antonio Modesto, Marcos Amador, Camile Lopes, Sílvia Helena Rabenhorst, Raquel Montenegro, Bruno D. A. Souza, Thayson Lopes, and Vivian Silbiger did not respond to correspondence from the Editor about this Expression of Concern.

